# Context aware benchmarking and tuning of a TByte-scale air quality database and web service

**DOI:** 10.1007/s12145-021-00631-4

**Published:** 2021-06-07

**Authors:** Clara Betancourt, Björn Hagemeier, Sabine Schröder, Martin G. Schultz

**Affiliations:** Jülich Supercomputing Centre, Forschungszentrum Jülich, Germany

**Keywords:** Air quality data, Scientific database, Scientific web service, Performance tests

## Abstract

We present context-aware benchmarking and performance engineering of a mature TByte-scale air quality database system which was created by the Tropospheric Ozone Assessment Report (TOAR) and contains one of the world’s largest collections of near-surface air quality measurements. A special feature of our data service https://join.fz-juelich.de is on-demand processing of several air quality metrics directly from the TOAR database. As a service that is used by more than 350 users of the international air quality research community, our web service must be easily accessible and functionally flexible, while delivering good performance. The current on-demand calculations of air quality metrics outside the database together with the necessary transfer of large volume raw data are identified as the major performance bottleneck. In this study, we therefore explore and benchmark in-database approaches for the statistical processing, which results in performance enhancements of up to 32%.

## Introduction

Due to enhanced sensor technologies and widened monitoring efforts around the world, scientific databases of environmental observations have grown to terabyte scale. This can pose challenges on their performance, especially when the database is continuously extended with new data (Directorate-General for Communication EC 2018; Gray and Szalay 2002).

In this paper we present context-aware benchmarking and performance engineering of the Tropospheric Ozone Assessment Report (TOAR) data infrastructure, a terabyte (TByte)-scale scientific air quality database and connected web service. We apply the definition of context-aware given by Dey (2001): *”A system is context-aware if it uses context to provide relevant information and/or services to the user, where relevancy depends on the user’s task.”*.

The TOAR data infrastructure was created by the research centre Jülich in the context of the global TOAR initiative Schultz et al. (2017). It meets special requirements of the TOAR user community in terms of data acquisition, openness, functionality, flexibility, performance and FAIRness (Wilkinson et al. 2016). Due to its extensive and flexible on-demand processing capabilities, it offers a novel kind of scientific data service. The design of such services must be guided by expertise in earth system science and computer science to ensure a user-friendly and trusted application with adequate performance. While our online data services have been good enough for the analysis of up to 100 individual time series, we are aware of performance bottlenecks when it comes to larger data queries. Although the general topic of database performance is well discussed in the technical literature, we found no published case studies on tuning scientific databases. This motivated us to study various potential performance improvements and report them here.

## Performance of scientific database applications

Making database applications fast has become a hot topic, since rapid data availability and processing are a limiting factor of end-user data services and related applications when databases grow to TByte-scale and beyond.There exist various tuning approaches for general database management systems (DBMS) and connected (web-)services. Logical database tuning methods like scheme enhancements and denormalization (Westland 1992) may require extensive reorganization of the database, possibly involving longer downtimes (Thalheim and Tropmann-Frick 2011). Tuning by adjusting performance critical parameters of the database is often difficult due to the complexity of the system and large number of parameters, which might influence different database applications in different ways. There are a number of tuning advisory frameworks (e.g. Lu et al. (2019)) that can support this process. Physical tuning methods like indexing and query optimization are well understood in general (Shasha and Bonnet 2004; Thalheim and Tropmann-Frick 2011). However, evaluation and improvement of DBMS performance in real world applications is only possible when also taking the user perspective into account. This means in practice, that tuning will be most successful if the entire data service including the underlying database(s) and (web-) services are treated as a whole and tuned in a context-aware approach (Dey 2001; Nimalasena and Getov 2014). A deep understanding of the use cases and bottlenecks of DBMS and their applications has led to various innovative solutions in the data science field (Kersten et al. 2011; D’Silva et al. 2019; Sandha et al. 2019).

Fast access to scientific data products increases the quality, flexibility and outreach of scientific workflows, for example by enabling researchers to investigate different scenarios and view more data. As such, the availability of air quality data products enabled the rapid publication of numerous studies which assess changes in air quality during the COVID-19 lockdowns in the year 2020 (see e.g. Farahat et al. 2021; Gkatzelis et al. 2021). Fast access to scientific data products becomes especially important in interdisciplinary research contexts. As an example, the TOAR data, which were assembled and analyzed by atmospheric scientists, are increasingly used by medical researchers to investigate impacts of air pollution on human health (Stanaway et al. 2018). Good performance of a scientific DBMS is achieved e.g. by adapting indices, buffer size, and parallel processing. The most frequent queries are monitored and can be tuned if necessary. This is a continuous process since the database might grow, and frequent query patterns might change over time. Physical performance monitoring can be supported by query plans and tuning advisory frameworks. Furthermore, common relational databases might not be ideal for processing time series since they do not take advantage of the order of rows (Shasha and Bonnet 2004). This pushed the recent development of (often partly closed source) time series databases (InfluxData et al. 2018; Nasar and Kausar 2019).

Additionally, large scientific databases and connected applications face typical context-related performance bottlenecks. Scientific data is often stored in a relational database, but is required to be in non-relational format for analysis and further processing. This so called ”object-relational impedance mismatch” (Ireland et al. 2009) often degrades the performance of the database applications. It is common to query relational data from a scientific database and transfer it to another system, where the data is converted to non-relational format and analyzed. The performance of this workflow is diminished by two factors: 1) Transfer of large amounts of data from one system to another 2) Converting relational to non-relational data representation. The transition from relational to non-relational format is unavoidable in some cases, but large data transfers can be averted when (parts of) the data processing is carried out inside the DBMS. Processing then happens ”near the data” on the database server. This concept was adapted e.g. by D’Silva et al. (2019), who developed an in-database interactive data exploration framework based on Python, which offers more flexibility than user defined in-database functions and a better performance than external data science frameworks. In another approach, Sandha et al. (2019) train a machine learning algorithm with big data input inside a DBMS using the Teradata SQL engine.

In practice, the workflow of querying data from a database, and processing it for further analysis is always a trade-off between performance and flexibility, the two extremes being user defined functions with minimum flexibility and maximum performance on the database side and pure outside-calculations on the client side. Furthermore, obtaining raw data and developing own applications may be impracticable for the scientific end user, when programming takes a long time as the needed analysis is complex, or when calculations might not be easily reproducible by others. This can be overcome by connecting the scientific DBMS to a (web)-service that offers on-demand calculation of well documented data products which meet the needs of the scientific community in that specific field. There exist some large scientific database providers which link the DBMS to a web-API that offers basic standardized functionality like averaging over an axis or cutting out a domain window, e.g. in astronomy and earth science (Wagemann et al. 2018; Bereta et al. 2019; Gray and Szalay 2002). The database described in this paper goes one step further and offers more sophisticated air quality metric data products as part of an open web-service.

## TOAR database and data services

TOAR created one of the world’s largest databases for near-surface air quality measurements (Schultz et al. 2017). Data in the TOAR-DB have been collected from different public bodies, air quality networks, and research institutions all over the world, and the database continues to grow. More than 350 users from 35 countries have accessed the TOAR-DB via the graphical web interface JOIN^1^ or the REST API^2^ and downloaded station information and aggregated statistics of ozone and associated variables. All statistics are calculated online from the hourly data that are stored in the database to allow for maximum user flexibility. These statistics were used in the first Tropospheric Ozone Assessment Report, which was published as a series of peer-reviewed journal articles (Fleming et al. 2018; Gaudel et al. 2018; Lefohn et al. 2018; Chang et al. 2017; Young et al. 2018; Mills et al. 2018; Tarasick et al. 2019; Xu et al. 2020). The data were also analyzed for the 2017 Global Burden of Disease assessment (Stanaway et al. 2018).

**Fig. 1:**
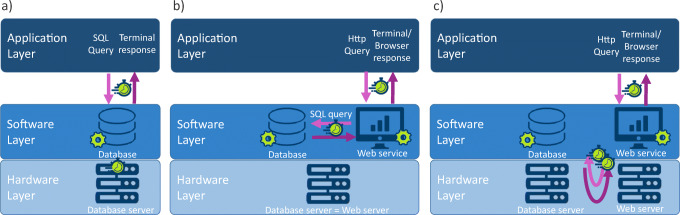
Configurations of database-driven data services: **a** Direct access to the database via SQL commands. **b** The database is accessed via a web service API; both service components run on the same hardware. **c** Our configuration as described in TOAR database and data services: as **b**, but the two service components operate on different hardware. The gear wheels and stop watches denote performance-critical data processing and data transfer

The TOAR-DB is a PostgreSQL V10 relational database. PostgreSQL was chosen because it is open source, widely used in the scientific community and highly scalable. The database server and JOIN web server are located on virtual machines (VM) on different hosts inside an OpenStack cloud environment at Jülich Supercomputing Centre (JSC). The physical location of the data (tablespace) is mounted via NFS over a 10 GBit/s connection. The server side system is an IBM Spectrum Scale with a total storage of 52PByte (Jülich Supercomputing Centre 2019b). The physical systems running the VMs are Fujitsu Primergy RX2530 M4 servers each equipped with 384 GByte of RAM and two Intel Xeon Gold 6126 12-core processors (Jülich Supercomputing Centre 2019a). The VM hosting the database is equipped with four VCPUs and seven GByte of memory. In the current set-up the web services trigger SQL queries via the Python psycopg2 library on demand and the resulting raw data are transferred to the JOIN server and processed locally to derive the statistical quantities requested by the end-user. It is evident that obtaining data products with this setup entails more performance-critical steps than simple command-line access to a database. In Figure 1 we therefore show performance-critical processes in various possible configurations of database-driven data services, including ours.

The TOAR-DB model largely follows the 3rd normal form. It consists of four tables with metadata information (variables, networks, stations, timeseries) and separate data tables for each variable (e.g. ’o3_hourly’ for hourly O_3_/ozone mixing ratio data). All data values are stored as individual rows in the data tables. The data columns are timeseries_id, timestamp, value, and two status columns. The primary key of the data tables is the combination of timeseries_id and timestamp. For example, the ozone table consists of approximately 10^9^ entries. Individual time series can be up to 40 years long, resulting in up to 350,000 values per time series.

The most common query patterns on the large data tables are (i) extraction of individual timeseries to calculate aggregate statistics or air quality metrics, and (ii) extraction of all values at a given timestamp to generate maps. Furthermore, it occurs relatively frequently that users want to see the highest (or lowest) N values of a given variable, possibly restricted to a given time period. To maximize performance in all of these cases, indices were defined for timestamp, value, and the combination of timestamp, value and timeseries_id on the ozone table, because it is used most frequently.

The web API to the TOAR-DB is written in Python-Django (with NumPy, SciPy and pandas packages for numerical calculations (van der Walt et al. 2011; Virtanen et al. 2020; McKinney 2010)) and offers more than 30 metrics that are commonly used by the international ozone research community and air quality agencies. Air quality metrics are used to consolidate air quality information from longer time periods into a single figure, which is then used for decision-making and air quality assessment. There exist different metrics for different air quality impacts, e.g. on human health or crops and vegetation. For instance, the AOT40 vegetation metric cumulates ozone exceedances above 40 parts per billion (ppb), assuming that vegetation is only harmed by ozone concentrations above that threshold. Data capture criteria are applied in the metric calculations to provide robust figures for decision making. For example, to calculate the European ozone standard,^3^ daily maximum 8-hour average concentrations have to be calculated and the 26th highest value must be reported under the condition that all days entering the calculation have at least 18 hours of valid measurements. Thus many metrics contain combinations of filtering and aggregation. Documentation on all metrics is available in Schultz et al. (2017).

## Benchmarks

The primary objective of this work is to compare the performance of in-database data processing versus calculations on the web server to find out if it is worthwhile to refactor the existing TOAR-DB and web service codes. To thoroughly understand performance, we designed several benchmarks, each highlighting a specific aspect, such as (parallel) data processing, data transfer and indexing. Each benchmark consists of various test cases that solve the same task in different ways. For example, to understand the performance of calculating air quality metrics inside vs. outside the database, we rewrite the Python/Pandas routines to SQL, and check if we save time by doing the calculation inside the database instead of Python. During a benchmark, test cases are repeated several times while the average performance is monitored. Most benchmarks are carried out on the ozone table as this is a blueprint for other tables in terms of structure, size and indexing. In the following, we detail the benchmarks and the different test cases they consist of. A summary is given in Table 1.

**Table 1:** Benchmarks summary

Benchmark	Tasks	Test cases
Data aggregation inside versus outside the database	Aggregation of ozone values for given dates: - Count - Maximum - Average - Standard deviation	1) ’Python’ 2) ’SQL’ 3) PL/Pythonu
Data aggregation inside versus outside the database	Ozone metrics for given series identifiers and years: - drmdmax1h - AOT40 - dma8epa - W90	1) ’Python’ 2) ’SQL’
Parallel processing	Processing of full ozone table: - Parallel scan - Parallel aggregate	1) max. 1 worker 2) max. 2 workers 3) max. 4 workers 4) max. 8 workers
Influence of indices on query times	Aggregation of values for given dates: - Maximum value on a given date	1) ’o3_hourly’ 2) ’temp_hourly’
Transfer times between database server and web server	Test bandwidth and latency	1) Ping round trip time test 2) Bandwidth test

For elaboration, see text

### Data aggregation inside versus outside the database

This benchmark aims to compare the performance of different aggregate functions inside the database in SQL versus outside the database in Python. Pure aggregation of data is more basic than use-case calculations for our web service, but its performance generalizes to more complex applications. The task for this benchmark is the aggregation of ozone data from all available stations, filtered by a randomly selected single day between the years 2000 and 2010. The date is chosen randomly to avoid caching and thus influencing the query time. The temporally filtered sub sets consist of approx. 10^5^ entries each. The aggregation was performed on these sub sets of ozone values. Four different aggregates were applied, covering a variety of numerical complexities and reflecting the usual data processing in scientific analysis: counting entries (’count’), finding the maximum entry (’max’), the mean value of all entries (’avg’) and their standard deviation (’std’). For each aggregate, three different test cases were compared against each other: 1) ”Python”: The temporally filtered data was queried from the database and further aggregated in the Python data science framework NumPy outside the database. 2) ”SQL”: The data was filtered and subsequently aggregated in form of a single SQL query. 3) ”PL/Pythonu”: The aggregate of filtered data was calculated inside the database by a user defined function in the imported procedural language PL/Pythonu. For every test case, ozone data from 100 randomly selected dates were aggregated, and the calculation times were monitored to obtain the average performance and its stability. This benchmark was conducted entirely on the database server, even when the data was processed outside the database, to avoid additional instability and bias through transfer times between different machines. Transfer times were tested individually (see benchmark Transfer times between database server and web server below).

### Metrics calculation inside versus outside the database

The main performance-critical use cases of our data service infrastructure benchmarked in this study are the calculation of different annual air quality metrics calculations on the ozone data table inside vs. outside the database. So far, the web service queries raw hourly ozone data from the database, transfers them to the web server, and calculates the metrics in the Python-Pandas data science framework. Here we test the performance that is gained if the calculation is instead conducted inside the database in SQL. We selected four different metrics that reflect different typical numerical patterns in the calculation: ’drmdmax1h’, which involves a daily maximum and rolling mean, ’AOT40’ which accumulates values above 40 ppb and applies a data capture criterion, ’dma8epa’ which additionally applies an hourly rolling mean and ’W90’ which involves an exponentially weighted hourly rolling mean. For an exact description of the metrics calculation, please refer to the Appendix. Random time series identifiers and random years between 2005 and 2010 were selected for the metrics calculation to avoid caching. For every metric, two test cases were compared against each other: 1) ”Python”: Hourly ozone data from the given time series and period is queried from the database, and further processed in Python-Pandas. 2) ”SQL”: The calculation was rewritten in a user defined function in SQL, and performed inside the database. For every test case, metrics for 250 randomly selected time series and years were aggregated, and the calculation times were monitored to obtain the average performance and its stability. As in the previous benchmark, transfer times between machines were avoided by Python processing on the database server.

### Parallel processing

In contrast to the two previous benchmarks, here we examine database speedup via parallel scans and aggregation in the database. The speedup is tested by varying the maximum number of parallel workers allowed in a query. The number can be set in the system parameters of the database. Two tasks were designed, which allow effective parallel processing. ’scan’: A parallel index scan across the entire ozone table that filters all ozone values below zero, indicating incorrect values. ’agg’: A parallel scan and aggregate across the full ozone table to output the mean of all ozone values. Four test cases were compared against each other: 1) Allowing 1 parallel worker, 2) allowing 2 parallel workers, 3) allowing 4 parallel workers 4) allowing 8 parallel workers. For each test case, the time taken to complete the tasks was monitored. For better comparability, all test cases were executed with cold system cache.

### Influence of indices on query times

With this benchmark we examine the importance of indices for the performance of temporal filtering. Air quality monitoring data consists of time series, so many of our database queries require filtering over time. We test the performance gained from setting useful indices by querying temporally filtered data from two tables that are similar structure, but have different indices. First, the ozone table, which was already used in the previous benchmarks and has an index on timestamp, value and timeseries_id in addition to the primary key on timeseries_id and timestamp. Second, the slightly smaller temperature table, which contains hourly temperature values and has only the primary key on timeseries_id and timestamp. The task to fulfill here corresponds to benchmark Data aggregation inside versus outside the database, test case 2) ”SQL”: query all data from a randomly selected date between 2000 and 2010, and output the maximum value. Two test cases were compared against each other: 1) ”o3_hourly”: The task was performed on the ozone table. Here we reused the results from benchmark Data aggregation inside versus outside the database. 2) ”temp_hourly”: The task was performed on the temperature table. Since the query times showed little deviation from the mean, we only performed 20 queries for this test case.

### Transfer times between database server and web server

As described in TOAR database and data services, DB server and web server are VMs on different hosts. This means that transfer between the machines is not avoidable. Yet, by moving data processing to the database server, time is saved not only by processing in SQL instead of Python, but also by reduced data transfer between machines. Transfer times between the database server and web server are tested independently of the DBMS. To estimate the latency, the Ping round trip time was tested. The bandwidth is theoretically 10 GBit/s (see TOAR database and data services), but might be lower in every day use. It was tested with the iperf tool^4^.

## Results

**Fig. 2:**
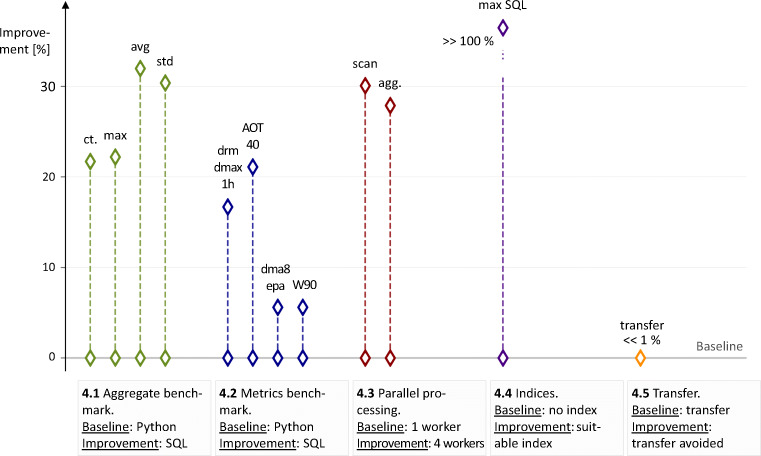
Graphical summary of the benchmark results. We give the ideal configuration for each benchmark and compare the relative performance enhancement with the baseline case

In the following Subsections, we present the results of the benchmarks described in Benchmarks. A graphical summary is given in Figure 2.

### Data aggregation inside versus outside the database

The results of benchmark Data aggregation inside versus outside the database are summarized in Table 2. The mean absolute query times vary between 0.16 and 0.27 seconds. They are relatively stable with a standard deviation of 0.02 to 0.08 seconds for 100 queries. SQL aggregates are always the fastest, with relative time savings of 21.7% to 32.0%. It is noticeable that when comparing Python and SQL directly (last column in Table 2), less time is saved with less computationally complex calculations (’count’) and more time is saved with more complex the calculation (’avg’, ’std’).

**Table 2:** Benchmark Data aggregation inside versus outside the database results

Aggre -	’Python’	’SQL’	’PL/Python’	Difference
gate	*μ* ± *σ* [s]	*μ* ± *σ* [s]	*μ* ± *σ* [s]	’Python’-
				’SQL’
’count’	0.23 ± 0.05	**0.18**±**0.08**	0.22 ± 0.03	21.7 %
’max’	0.27 ± 0.05	**0.21**±**0.07**	0.26 ± 0.07	22.2 %
’avg’	0.25 ± 0.04	**0.17**±**0.03**	0.26 ± 0.03	32.0 %
’std’	0.23 ± 0.04	**0.16**±**0.02**	0.27 ± 0.06	30.4 %

The cells contain execution time and standard deviation (n = 100 each). The fastest aggregation is marked in bold

### Metrics calculation inside versus outside the database

The results of benchmark Metrics calculation inside versus outside the database are summarized in Table 3. The metrics calculation times range between 0.15 and 0.19 seconds, with a low standard deviation of 0.02 seconds maximum. Calculating metrics in SQL is always faster than Python. The time difference varies between 5.6 % and 21.1 %. In SQL, metrics that include the calculation of hourly rolling means/sums (’dma8epa’, ’W90’) are comparatively slower than metrics that include only aggregates (’drmdmax1h’, ’AOT40’). For the calculation of metrics within the database, the SQL query plan showed that approx. 50 % of the query execution time is spent on the selection of time-filtered raw data and 50 % on the processing (windows, aggregates, data capture filtering) of these data. The planning time was negligible (< 1 % of the query execution time).

**Table 3:** Benchmark Metrics calculation inside versus outside the database results

Metric	’Python’	’SQL’	Difference
	*μ* ± *σ* [s]	*μ* ± *σ* [s]	
’drmdmax1h’	0.18 ± 0.01	**0.15**±**0.01**	16.7 %
’AOT40’	0.19 ± 0.02	**0.15**±**0.02**	21.1 %
’dma8epa’	0.18 ± 0.02	**0.17**±**0.02**	5.6 %
’W90’	0.18 ± 0.02	**0.17**±**0.02**	5.6 %

The cells contain execution time and standard deviation (n = 250 each). The fastest metrics calculation is marked in bold

### Parallel processing

The results of benchmark Parallel processing are summarized in Table 4. When two or four workers are allowed instead of one, the query time is reduced by approx. 20-30 %. Even though the best results were obtained with four allowed workers, it is noteworthy that the difference between one and two workers is quite large, while adding two more workers does not save much more time. If eight workers are allowed, no more time is saved because of the parallel overhead.

**Table 4:** Benchmark Parallel processing results

	1 worker	2 workers	4 workers	8 workers
Task	allowed	allowed	allowed	allowed
	[#] / [s] / [%]	[#] / [s] / [%]	[#] / [s] / [%]	[#] / [s] / [%]
	1	2	4	5
’scan’	0.93	0.68	**0.65**	0.98
		- 26.9 %	**- 30.1**	+ 5.4 %
	1	2	4	8
’agg’	99.03	78.71	**71.43**	76.10
		- 20.5 %	**- 27.9 %**	- 32.2 %

The cells contain the actual number of workers spawned, the execution time, and the difference to execution time with one worker. The best result for each task is marked in bold

The query planner considers time needed to spawn workers, process in parallel and gather results. Depending on the query, it may thus be infeasible to spawn all workers which are allowed. E.g. for the ’scan’ test case: when eight workers are allowed, only five workers are spawned. Here it is notable that the query takes longer than in the case of one allowed worker. This points to a need for improvement in the planners’ cost constants, which would require extensive experimenting (The PostgreSQL Global Development Group 2015).

During the parallel processing benchmarks, the CPU load was below 25 %, indicating that the performance in this case is I/O bound. This is another explanation why the addition of more workers does dot necessarily result in a better performance.

### Influence of indices on query times

The results of benchmark Influence of indices on query times are summarized in Table 5. The scan on the ’o3_hourly’ table used the index on timestamp, value and timeseries_id, which allows fast temporal filtering. the ’temp_hourly’ table has only the primary key on id and timestamp, so here a sequential scan was required for temporal filtering, which took several orders of magnitude longer. Since the ’temp_hourly’ table is slightly smaller than the ’o3_hourly’ table, this provides a lower limit for the drop in performance with sub-optimal indices.

**Table 5:** Benchmark Influence of indices on query times results

	’o3_hourly’	’temp_hourly’
Aggregate	(n = 100)	(n = 20)
	*μ* ± *σ* [s]	*μ* ± *σ* [s]
’max SQL’	**0.21 ± 0.07**	82.63 ± 2.41

The fastest aggregation is marked in bold

### Transfer times between database server and web server

The results of benchmark Transfer times between database server and web server are summarized in Table 6. The data transfer rate between the database server and the web server is theoretically 10 GBit/s. With a measured bandwidth of 8.3 GBit/s, approx. 80 % of this maximum transfer rate is reached during tests. For a typical metric query, which requires hourly timestamp/value pairs of 10 years for processing, about 1.2 MByte of data is transferred from the database server to the web server. This means that the transfer time for calculations outside the database is less than 1 ms. This makes less than 1 % of the required calculation time and is therefore negligible. The ping round trip time was measured to be 0.7 ms, and is therefore not adding substantially to the query times.

**Table 6:** Benchmark Transfer times between database server and web server results

Transfer	Bandwidth	Latency
Between VMs		0.7 ms
in OpenStack	8.3 GBit/s	ping round
cloud environment		trip time

These results show that due to the high bandwidths and the relatively small amount of data transferred, the transfer time between the different machines is not a limiting factor for our setup. This may be different for other applications where larger amounts of data need to be transferred, such as multi-dimensional geospatial data instead of one-dimensional time series. This case would require more detailed benchmarking and monitoring of transfer times.

## Conclusion

We benchmarked the following measures to increase the performance of the TByte-scale TOAR air quality observations database and connected JOIN web service: server-side programming in PL/pgSQL and PL/Python, parallel scans/processing, optimal definition of indices, and on-line aggregation to avoid transfer of large data. Through the above mentioned techniques, the performance of JOIN can be improved in a range of approx. 6–32%.

Comparing the benchmark results, it becomes clear that in-DB data processing saves more time by switching from Python to SQL (up to 32%), than by avoiding data transfer or planning times (≪ 1%). Calculations of air quality metrics that do not require moving averages perform significantly better in SQL than in Python (approx. 17-21%). Yet, when rolling averages are required, which forces the use of window functions, the advantage of SQL to Python is diminished to approx. 6 %. When thinking about replacing the existing Python code with SQL queries an important aspect to consider might be that Python functions are easier and more flexible to program than SQL user defined functions. Our findings regarding in-database processing are thus comparable to D’Silva et al. (2019), who state that in-database processing allows performance increase, yet common user defined functions on the database server are more difficult to implement than client side programming in Python. We can generalize this statement from scientific databases to typical data structures used for environmental data. Like other database systems, a prerequisite for good database performance of TOAR and its connected services is a suitable physical DB-design and a good technical infrastructure. Our current setup on the OpenStack cloud environment with a fast connection, the physical DB design, and a web service which is tailored to the needs of the scientific user community has proved its worth. To exploit advantages of parallel processing, more extensive tuning of cost parameters would be necessary. We do not see this as worthwhile because the performance of our DB is I/O-bound and our most common query patterns cannot be improved by parallel processing. Our DB scheme is expected to scale well to expected database growths, while maintaining a good performance.

We expect our findings to generalize well to comparable database systems and web services on cloud systems. This study as an example of systematic exploration of performance aspects in a mature environmental database and web service is thus of interest to the growing community of scientists who aim to make scientific data products openly available. Big data applications are getting more and more attention in the field of environmental science, yet we have found few technical literature on database structures containing atmospheric observation data. This study also shows the importance of collaboration between domain scientists and database engineers/computer scientists when the performance of scientific databases shall be analysed and improved. Without the detailed specification of real-life query patterns (e.g. calculations including a rolling mean) the benchmark tests might easily become meaningless. Due to rapidly evolving demands from scientists on the database design and capabilities and usually limited resources for optimisation, a balance must be found between best possible performance and a reasonable optimisation effort. Nevertheless, this study proves that such analysis can be worthwhile and we hope that our specific benchmarking tests can provide useful hints to others where to look first. Finally, we give our ranking of important aspects for a well-functioning, high-performance database and web service for the scientific community: 1) Well documented data products tailored to the needs of scientists, 2) A context-aware DB infrastructure and physical DB design (e.g. indexing), 3) In-DB data processing in SQL instead of on the web server in Python.

## Appendix: Air quality metrics description

**Table:**

Metric	Application	Description
drmdmax1h: Maximum 3-month average of the daily 1-h maximum ozone value (Brauer et al. 2016)	Human health	3-months running mean of daily maximum 1-hour mixing ratios are calculated. For annual statistics, the maximum value will be computed.
AOT40: Cumulative exceedance of 40 ppb ozone concentration (Mills et al. 2017)	Vegetation	Daily 12-h AOT40 values are accumulated using hourly values for the 12-h period from 08:00h until 19:59h solar time interval. AOT40 is defined as cumulative ozone above 40 ppb. If less than 75 % of hourly values (i.e. less than 9 out of 12 hours) are present, the cumulative AOT40 is considered missing. When there exist 75 % or greater data capture in the daily 12-h window, the scaling by fractional data capture (ntotal/nvalid) is utilized. For monthly, seasonal, summer, or annual statistics, the daily AOT40 values are accumulated over the aggregation period and scaled by (ntotal/nvalid) days. If less than 75 % of days are valid, the value is considered missing.
dma8epa: The 4^th^ highest daily maximum 8-h average (Federal Register 2015)	Human health	Daily maximum 8-hour average statistics according to the US EPA definition. 8-hour averages are calculated for 24 bins starting at 00:00h local time. The 8-h running mean for a particular hour is calculated on the concentration for that hour plus the following 7 hours. If less than 75 % of data are present (i.e. less than 6 hours), the average is considered missing. A daily value is considered valid if at least 18 hourly averages are valid. For annual or seasonal statistics, the 4^th^ highest daily 8-hour maximum of the aggregation period will be computed.
W90: 4th highest W90 5-h cumulative exposure index (Lefohn et al. 2010)	Human health	EI = SUM(w_*i*_*C*_*i*_) with weight w_*i*_ = 1/[1 + M exp(-A C_*i*_/ 1000)], where M = 1400, A = 90, and where C_*i*_ is the hourly average O_3_ mixing ratio in units of ppb. For each day, 24 W90 indices are computed as 5-hour sums, requiring that at least 4 of the 5 hours are valid data (80 %). If a sample consists of only 4 data points, a fifth value shall be constructed from averaging the 4 valid mixing ratios. For seasonal or annual statistics, the 4^th^ highest W90 value is computed, but only if at least 75 % of days in this period have valid W90 values.

## Footnotes

1 https://join.fz-juelich.de

2 https://join.fz-juelich.de/services/rest/surfacedata/

3 https://ec.europa.eu/environment/air/quality/standards.htm

4 https://iperf.fr/iperf-download.php
